# Mechanosensitivity in the enteric nervous system

**DOI:** 10.3389/fncel.2015.00408

**Published:** 2015-10-13

**Authors:** Gemma Mazzuoli-Weber, Michael Schemann

**Affiliations:** Human Biology, Technische Universitaet MuenchenFreising, Germany

**Keywords:** enteric nervous system, gut reflexes, mechanoreceptor, compression-sensitive, tensile-sensitive

## Abstract

The enteric nervous system (ENS) autonomously controls gut muscle activity. Mechanosensitive enteric neurons (MEN) initiate reflex activity by responding to mechanical deformation of the gastrointestinal wall. MEN throughout the gut primarily respond to compression or stretch rather than to shear force. Some MEN are multimodal as they respond to compression and stretch. Depending on the region up to 60% of the entire ENS population responds to mechanical stress. MEN fire action potentials after mechanical stimulation of processes or soma although they are more sensitive to process deformation. There are at least two populations of MEN based on their sensitivity to different modalities of mechanical stress and on their firing pattern. (1) Rapidly, slowly and ultra-slowly adapting neurons which encode compressive forces. (2) Ultra-slowly adapting stretch-sensitive neurons encoding tensile forces. Rapid adaptation of firing is typically observed after compressive force while slow adaptation or ongoing spike discharge occurs often during tensile stress (stretch). All MEN have some common properties: they receive synaptic input, are low fidelity mechanoreceptors and are multifunctional in that some serve interneuronal others even motor functions. Consequently, MEN possess processes with mechanosensitive as well as efferent functions. This raises the intriguing hypothesis that MEN sense and control muscle activity at the same time as servo-feedback loop. The mechanosensitive channel(s) or receptor(s) expressed by the different MEN populations are unknown. Future concepts have to incorporate compressive and tensile-sensitive MEN into neural circuits that controls muscle activity. They may interact to control various forms of a particular motor pattern or regulate different motor patterns independently from each other.

## The autonomous control of gut motility by the enteric nervous system

Neurons controlling gastrointestinal functions are located in a continuous ganglionated network within the gut wall. This autonomous system is referred to as the enteric nervous system (ENS). The name was coined by Langley in 1921 to acknowledge the ENS as a third division of the autonomic nervous system and to emphasize its functional independency from the central nervous system (Langley, 1921). This explains the nowadays often used alias “second (or little) brain in the gut” (Gershon, 1998). The ENS consists of two plexi, the myenteric plexus located between the two muscle layers and the submucous plexus just beneath the mucosal layer. The idea of an independency of the neuronal control of the gut from the central nervous system dated back to the middle of the nineteenth century when Lister (the pioneer of antiseptic surgery) described vermicular motion of the intestines even after complete division of the mesenteric nerves (Lister, 1857). The conclusion from this observation was later confirmed by the first detailed description of the local motility patterns (pendular movements and peristalsis) in the isolated or extrinsically denervated intestine (Bayliss and Starling, 1899, 1901). These studies reported that distension of the gut wall evoked a muscle contraction above and a relaxation below the distended area, a pattern referred to as the “peristaltic reflex.” It is noteworthy that even from Bayliss and Starling this pattern was not described as a stereotyped muscle response which can be consistently evoked. Very early on Magnus already pointed out that sometimes distension evoked contractions only (Magnus, 1904a,b,c). This has also been more recently confirmed with more sophisticated methods (Spencer et al., 1999). Moreover, the initiation of the “peristaltic reflex” varies with the speed of distension (Trendelenburg, 1917). A rapid distension readily evoked the reflex while the threshold was much higher with slow distension and this eventually failled to evoke the reflex. Trendelenburg concluded that muscle tone or resistance to distension due to elasticity of the smooth muscle layer impacts on the threshold to trigger peristalsis. It is likely that this control of muscle tone depends on nerves because the distension evoked muscle contraction above as well as at the site of stimulation was prevented by denervation (Lüderitz, 1890). The local increase in muscle tension just at the site of distension was viewed already at that time crucially important for the “peristaltic reflex.” More recently, elegant studies provided experimental evidence for the importance of the muscle tone for the initiation and maintenance of peristalsis along longer segments of the gut (Spencer et al., 2001; Smith et al., 2003). The “classic peristaltic reflex” should therefore be viewed as a pattern consisting of three components: an increased muscle tone at the site of distension, a contraction above and an inhibition of muscle activity below the distended region. To function independently and to generate muscle activity the ENS needs to possess neurons able to respond to mechanical stimuli and to initiate adequate motor responses. Moreover, the ENS needs to integrate signals that are generated by other nerves but at the same time must react to a constantly changing molecular composition of the micro milieu in the gut wall. Most mechanosensitive neurons involved in these reflex activities have to be located in the myenteric plexus because peristalsis persisted in preparations deprived of the mucosal-submucosal layer but ceased after removal of the myenteric plexus (Magnus, 1904a,b,c).

## Mechanical deformation of enteric neurons during muscle activity

**Figure 1:**
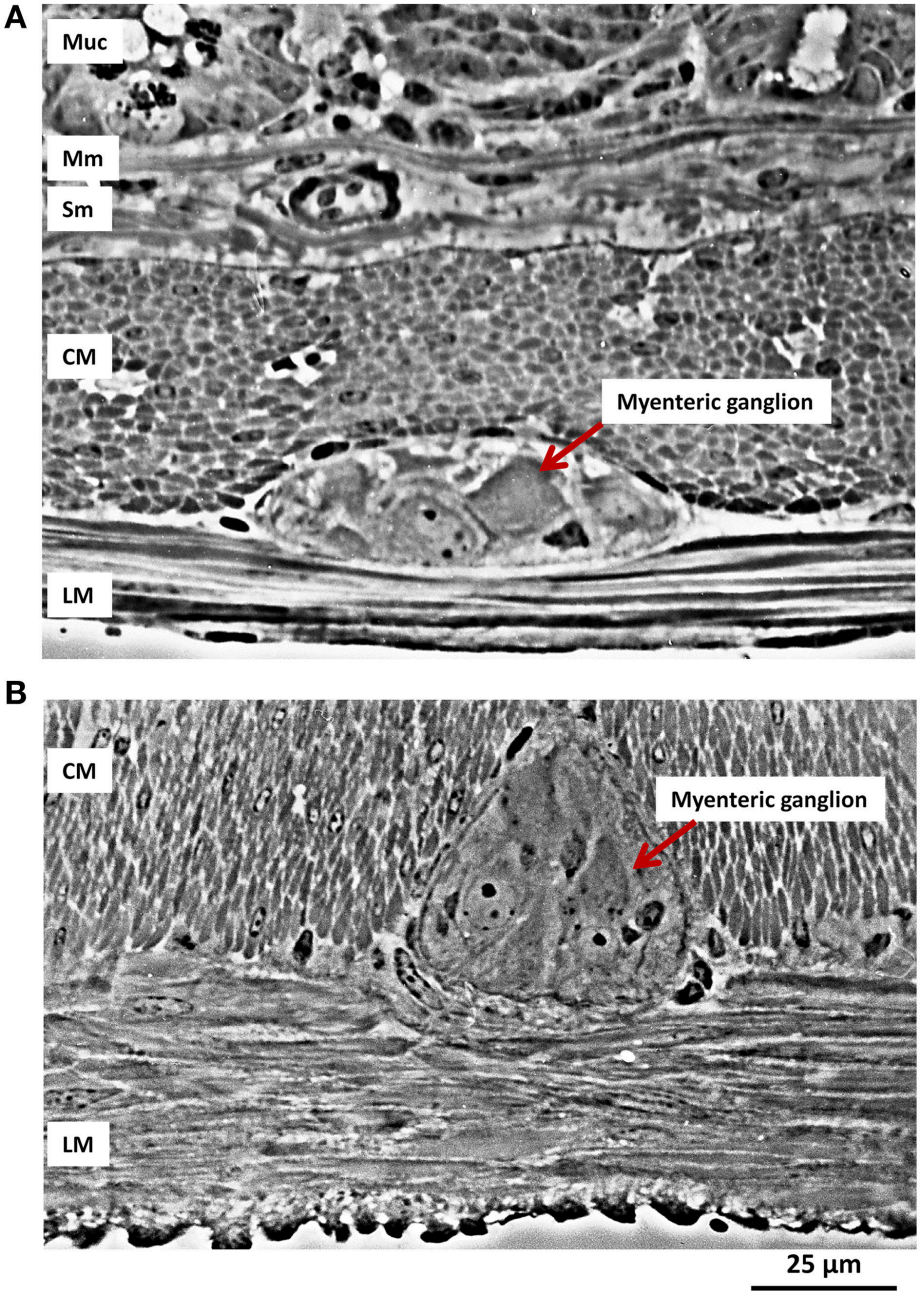
**Ganglionic deformation during muscle contraction**. **(A)** Transverse longitudinal section of the small intestine (ileum) of a guinea pig. The layers of the wall are shown in section, from the bottom part of the mucosa (Muc) with its glands, at the top, then the muscularis mucosae (Mm), the submucosa (Sub), with collagen bundles and a large blood vessel, the circular muscle (CM) layer, a myenteric ganglion and the longitudinal muscle (LM) layer. **(B)** In this preparation the longitudinal muscle is isotonically contracted, while the circular muscle layer is at rest; a myenteric ganglion is compressed sideways and pushed between bundles of circular musculature. (Micrographs kindly provided by Dr. Giorgio Gabella).

Neurons residing in the myenteric plexus are constantly deformed during muscle contraction and relaxation. This becomes obvious when viewing the deformation of a myenteric ganglion during muscle movements (Mazzuoli and Schemann, 2009). However, a quantitative assessment of ganglionic deformation in a contracted gut has been reported only once (Gabella and Trigg, 1984) (Figure 1). This study revealed considerable changes in neuronal shape with contraction and distension of the gut wall. The variations in cross sectional area may be as large as 95%.

**Figure 2:**
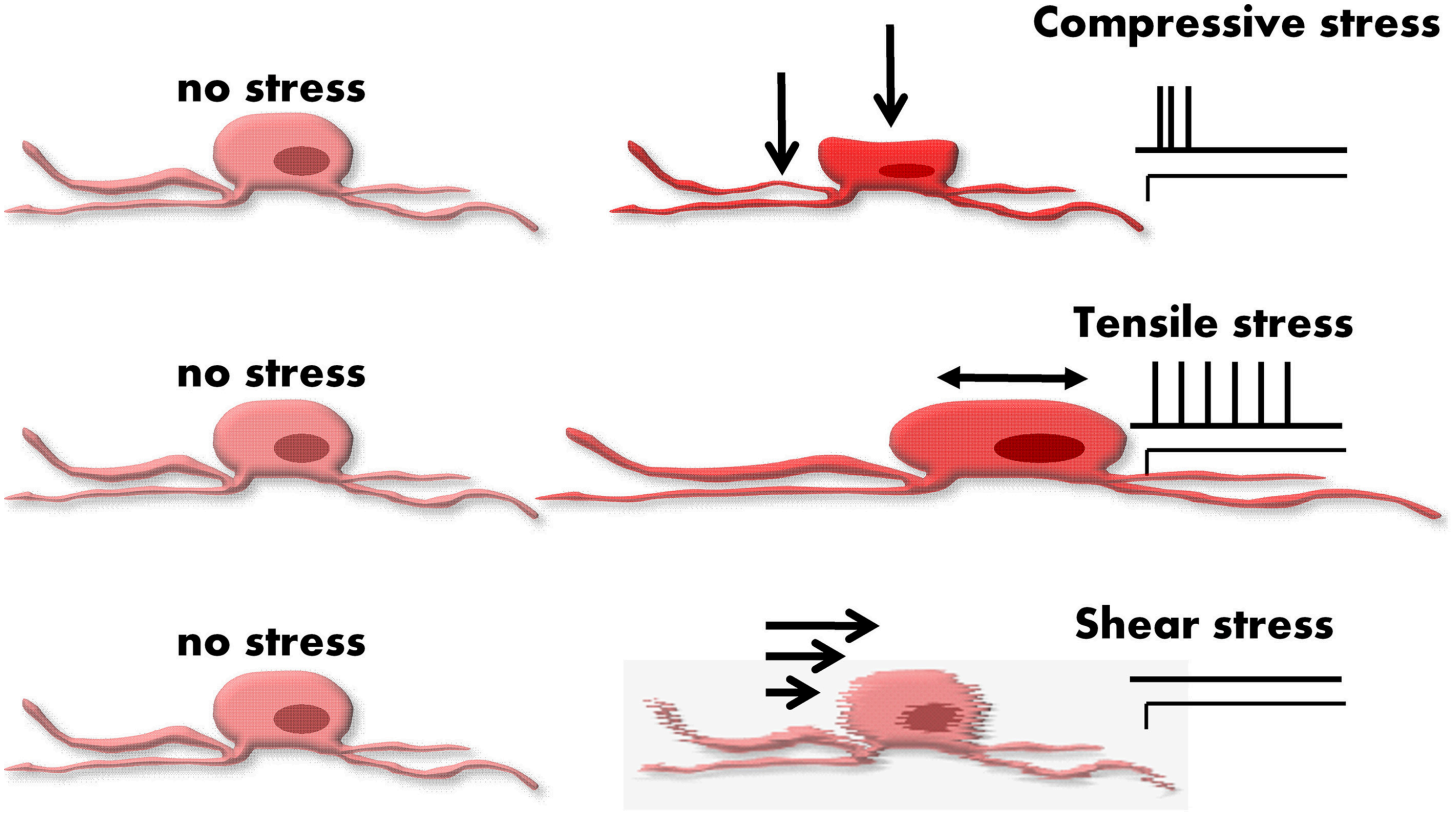
**Different types of forces acting on an enteric neuron**. From the top to the bottom: compressive, tensile, and shear stress. The different stresses evoke different deformation in the neuron. On the right side of the figure the spiking patterns of typical responses are drawn. Mechanosensitive enteric neurons (MEN) tend to respond with a rapidly adapting pattern to compression. A slowly or even ultra-slowly adapting pattern of firing often appears in response to tension. Shear stress hardly evokes any response and does not seem an important stimulus for MEN.

The deformation of a neuron during muscle activity is due to different types of mechanical stimuli (Gregersen, 2002). Strictly speaking, strain is the more accurate term than deformation. Strain is defined as relative changes in shape or size of a solid due to stress. Stress is proportional to strain and is defined as a force divided by an area. Although very simplified, we consider in this review three different types of stress representing the stimulus modalities often used in the field of biomechanics (Rajput, 2005) (Figure 2):

- Tensile stress—force that tends to stretch or lengthen a neuron—acts perpendicular to the stressed area.
- Compressive stress—force that tends to compress a neuron—typically compression goes along with volume decrease at the site of deformation—acts perpendicular to the stressed area.
- Shear stress—force tending to cause deformation of a material by slippage along a plane or planes parallel to the imposed stress.

One has to consider that it is currently not feasible to quantify such physical forces at a single cell level or to precisely classify the mechanical forces acting on individual cells (Liu et al., 1999). Neurons are elastic bodies and therefore length change in one plane will likely cause a length change in another plane. This means that there is never pure and isolated tensile stress without compressive stress and vice versa. It is much more realistic to classify mechanical forces acting on single cells by their primary stress modality. This review will therefore follow a rather pragmatic definition of mechanical forces acting on enteric neurons (Figure 2).

By now, a wealth of studies in different species has identified with different techniques enteric neurons responding to various types of mechanical stimuli (Table 1). The so far applied mechanical stimuli vastly differ between studies. The stress modalities were either compression using different types of von Frey hairs, tension by tissue stretch (with and without increased muscle tone) or a mixture of compression and shear stress evoked by intraganglionic volume injection. As discussed below, the compressive forces are most relevant during intraganglionic volume injection.

**Table 1:** **Properties of mechanosensitive enteric neurons (MEN)**.

**Reference**	**Species**	**Gut region**	**Identified by**	**Stimulus**	**Firing behavior**
Wood and Mayer, 1974; Mayer and Wood, 1975	Cat, dog, guinea pig	Duodenum/Jejunum	Extracellular recordings	Compression with a glass electrode (15–30 μm) or 20 μm platinum wire	RAMEN, SAMEN, USAMEN
Kunze et al., 1998, 1999	Guinea pig	Ileum	Intracellular recordings	Compression through muscle tone during circumferential Stretch (20–40%)	USAMEN (AH-IPANs)
Kunze et al., 2000	Guinea pig	Proximal duodenum	Patch Clamp	Compression with polished glass probe (20–80 μm) or a fine hair.	RAMEN, SAMEN (AH-IPANs)
Spencer and Smith, 2004	Guinea pig	Distal colon	Intracellular recordings	Tension by circumferential or longitudinal stretch	SAMEN, USAMEN (mechanosensitive interneurons)
Mao et al., 2006	Mouse	Ileum	Patch-clamp and intracellular recordings	Compression with von Frey hair	RAMEN, SAMEN (AH-IPANs)
Mazzuoli and Schemann, 2009	Guinea pig	Ileum	Voltage sensitive dye imaging	Compression with von Frey hair (0.3 and 2.7 mN) and intraganglionic volume injection	RAMEN, SAMEN, few USAMEN
Hibberd et al., 2012a	Guinea pig	Distal colon	Extracellular recordings	Compresion with von Frey hair (0.8–5 mN) and tension by circumferential stretch	USAMEN (viscerofugal neurons)
Mazzuoli and Schemann, 2012	Mouse	Ileum/colon	Voltage sensitive dye imaging	Compression by intraganglionic volume injection	RAMEN, SAMEN, few USAMEN
Dong et al., 2015	Rat	Esophagus	Ca^2+^ imaging	Cell swelling by application of hypoosmotic solutions	unknown
Kugler et al., 2015	Primary cultured guinea pig and human enteric neurons	Guinea pig ileum; human small and large intestine	Voltage sensitive dye imaging	Compresion with von Frey hair (0.4 ± 0.05 mN)	RAMEN, SAMEN, few USAMEN
(Mazzuoli-Weber and Schemann, in review)	Guinea pig	stomach	Voltage sensitive dye imaging	Compression by intraganglionic volume injection	RAMEN, SAMEN
				Tension by ganglionic stretch	SAMEN, USAMEN, very few RAMEN

There have been many years of debates about which enteric neurons can be considered as a sensory or primary afferent neuron. Those discussions rarely considered conceptual issues but rather engaged in semantics. The first important step to break this habit is to use a term which everyone in the field can accept: mechanosensitive enteric neurons—MEN. We will try to introduce a concept that merges different findings and classifies MEN according to the stimulus they primarily encode and to the firing pattern in response to different stress modalities. The firing behavior revealed rapidly, slowly and ultra-slowly adapting MEN (RAMEN, SAMEN, and USAMEN, respectively). In order to better define adaptation we calculated an adaptation index (Kugler et al., 2015).

$$\begin{array}{l} AI=\frac{\text{action potential frequency }500\text{ ms}-\text{end of the recording}}{\text{action potential frequency }0-500\text{ ms}} \end{array}$$

In our experiments the entire recording period was up to 5 s. According to their firing behavior we could distinguish three populations, RAMEN, SAMEN, and USAMEN (Kugler et al., 2015). The first one is made up of neurons which fire action potentials only during the first 500 ms after the beginning of the deformation and show a rapid adaptation (*AI* = 0). These neurons, according to our previous findings, were defined RAMEN (Mazzuoli and Schemann, 2009, 2012). The second population is made up of neurons having an *AI* > 0 and < 1, they were firing action potentials also after the first 500 ms after the stimulus onset but the firing rate is decreasing along the recording time. These neurons, also according to our previously published works were named SAMEN. The third population had an *AI* = 1. These neurons keep firing throughout the recording without any decrease in action potential frequency. They were named USAMEN.

For other stimuli or experimental protocols the time periods may change but a stringent definition of spike discharge adaptation *per se* helps to use more objective criteria. If not specified in the publications of others we concluded from the traces whether the MEN behaved as RAMEN, SAMEN, or USAMEN.

## MEN responding to shear stress

We cultured myenteric neurons in micro channels and stimulated them with different flow rates very similar to the ones used to identify shear stress evoked responses in endothelial responses. Those experiments did not reveal an important role of shear stress because only very few enteric neurons (~8%) fired at a very low frequency even at the highest flow rate. In other studies we used intraganglionic injection of a small volume of Krebs solution which exerted compressive and shear forces. We can exclude a major role of shear stress based on the following findings. While increasing compressive forces by longer duration of intraganglionic injection changed the response pattern of MEN, there was no change if the flow rate and thereby the shear forces were increased.

## MEN responding to compressive forces

The first studies showing that some myenteric neurons responded to compression were performed in the ‘70s (Wood and Mayer, 1974; Mayer and Wood, 1975). The authors used probes ranging from 15 to 30 μm in diameter to compress ganglia and observed three response patterns with extracellular electrodes. Some MEN behaved like slowly others as rapidly adapting mechanoreceptors. The third group of MEN responded with long-lasting trains of spikes 30–40 s in duration (Mayer and Wood, 1975). Although spiking in these neurons did not show adaptation during the recording time, we refer to them as USAMEN because each sensor will adapt at some point in time.

Another population of myenteric neurons responsive to compression was described by John Furness and colleagues. They identified myenteric neurons with particular electrophysiological and morphological properties in the guinea pig ileum called intrinsic primary afferent neurons (IPANs). IPANs were mostly multipolar neurons with a slow after-spike hyperpolarization (AH; Furness et al., 1998). To test mechanosensitivity a von Frey hair was used to stimulate soma or neurites of AH-IPANs (Kunze et al., 2000). AH-IPANs in guinea pig and mouse ileum fired soma action potentials when the neurites were probed (Kunze et al., 2000; Mao et al., 2006). The response to this compressive force was direct and independent of muscle tension. The authors did not specify the adaptation behavior but we conclude from the traces in the above cited papers that the spike discharge was in some rapidly and in others slowly adapting. In guinea pig and mouse ileum the responses to compression were independent of muscle tone because they persisted in the presence of muscle relaxants. Interestingly, AH-IPANs stopped firing when the soma was distorted by pressing down the recording electrode or by raising intra-electrode pressure (Kunze et al., 2000). So far inhibition of firing in MEN as a consequence of soma distortion has not been reported in other studies and the functional significance remains to be demonstrated.

Originally, AH-IPANs were described as stretch-sensitive neurons in the guinea pig ileum because they responded with ongoing spike discharge to sustained circumferential stretch 20–40% above slack (Kunze et al., 1998, 1999). This response however disappeared after the muscle was paralyzed, which argues in favor of a rather indirect effect because the neurons required an increased muscle tone to respond to tissue stretch. This suggests that these neurons actually respond to the compressive forces when muscle tone increased in a distended gut rather than to tensile forces. However, the different firing pattern of AH-IPANs after compression by von Frey hair probing (rather rapidly adapting) and circumferential stretch in a tissue with muscle tone (rather slowly adapting) remains a puzzle. Changes in strength and duration of compressive force are likely to affect adaptation. We observed this behavior in some compression-sensitive RAMEN which fired for longer periods when the stimulus strength was increased despite the still higher spike burst frequency at the beginning of the stimulus.

**Figure 3:**
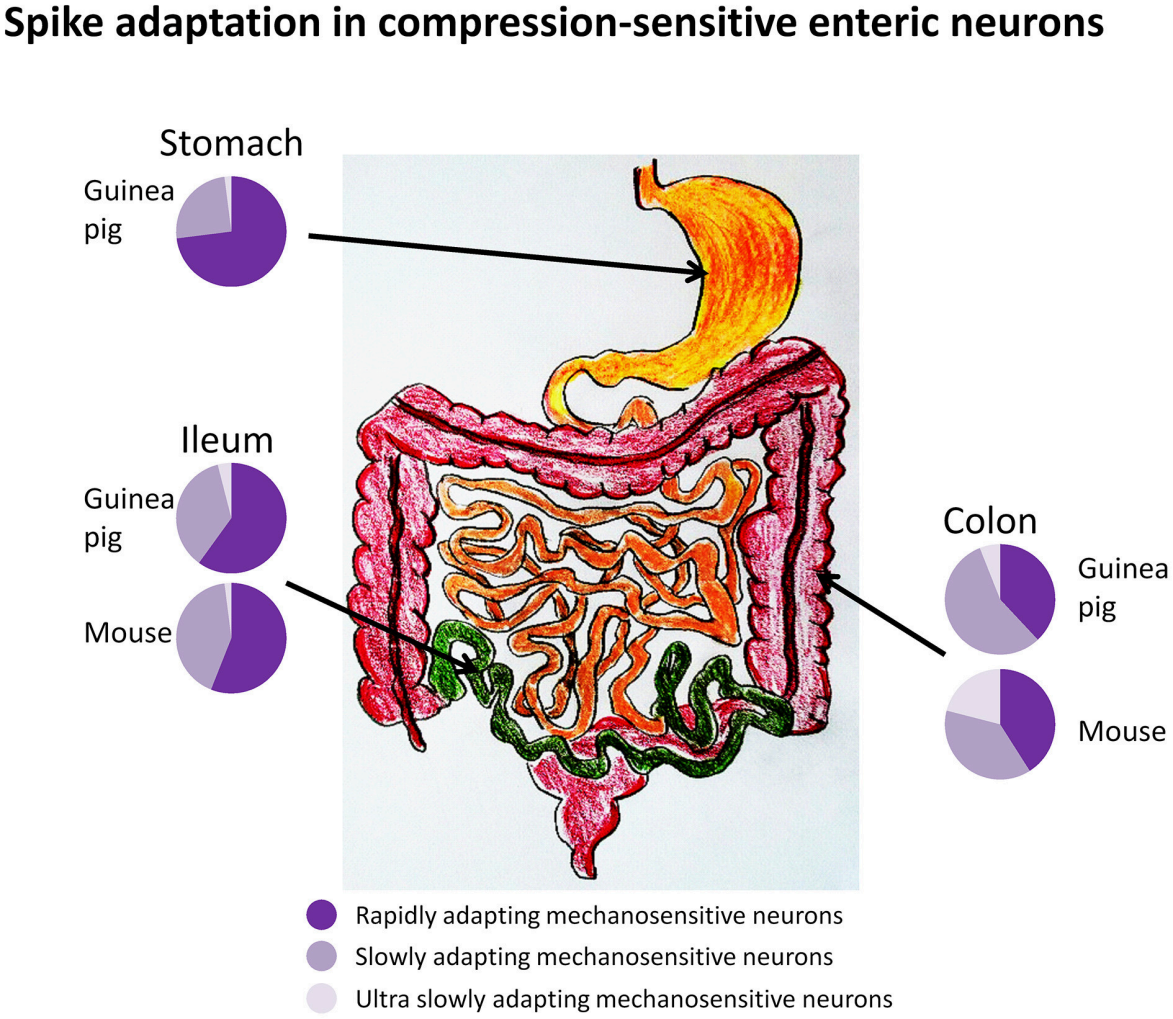
**Firing of compression sensitive MEN adapts rapidly, slowly, or ultra-slowly (RAMEN, SAMEN, or USAMEN, respectively)**. The proportion of RAMEN, SAMEN, and USAMEN varies along the gastrointestinal regions as illustrated by the pie charts.

Our group has presented the first systematic study on compression-sensitive MEN in guinea pig stomach, ileum and colon, mouse ileum and colon, and human intestine (Mazzuoli and Schemann, 2009, 2012; Schemann and Mazzuoli, 2010; Kugler et al., 2015; Mazzuoli-Weber and Schemann, in review; Figure 3). As a classical tool to induce compression we used von Frey hairs (Movie 1). The disadvantage of von Frey hairs was that we found it impossible to place the probe exactly onto the same spot after retraction. By chance we discovered that application of compressive forces onto enteric neurons can be achieved by intraganglionic injection of small volumes of fluid into a ganglion. The responses of MEN were identical between compression evoked by von Frey hair and intraganglionic volume injection (Mazzuoli and Schemann, 2009). The intraganglionic volume injection had the great advantage of evoking reproducible responses. Compression-sensitive MEN in our studies responded even after the muscle was paralyzed (Mazzuoli and Schemann, 2009, 2012). This was expected as we only distorted the ganglion and not the muscle. A more striking feature of compression-sensitive MEN was that not only interneurons but also circular muscle motor neurons responded to ganglionic compression (Mazzuoli and Schemann, 2009). Motor neurons were identified by tracing their projections to the muscle layer while processes of interneurons projected between ganglia. It was surprising to find that an equal proportion of interneurons and motor neurons responded directly, during synaptic blockade, to compression (Mazzuoli and Schemann, 2009, 2012). Firing of compression-sensitive MEN adapted rapidly, slowly, or ultra-slowly. Their proportion varied between the gut regions (Figure 3). RAMEN were dominant in the stomach but their proportion gradually decreased in more distal gut regions. Experiments conducted in primary cultured MEN revealed that they were more sensitive to neurite than to soma compression (Kugler et al., 2015). In addition, we found that many of their neurites conducted signals in both directions thereby serving afferent as well as efferent functions (Kugler et al., 2015).

An important feature of compression-sensitive RAMEN is their ability to encode changes in compressive forces independent of the resting (basal) mechanical stress (Mazzuoli and Schemann, 2009; Mazzuoli-Weber and Schemann, in review). While they adapt rather fast and stop firing even during sustained compression, they fire again a volley of action potentials if the compressive force is again increased. From this behavior it follows that RAMEN seem to reset during sustained distortion and respond again when they are exposed once more to the same degree of compression.

Conceptually, it is important to realize that, based on transmitter coding, morphology, synaptic and electrical properties and firing pattern after compression, most MEN described in our studies represent a population distinct from the AH-IPANs although both responded to compressive forces (Mazzuoli and Schemann, 2009, 2012). Only a few compression-sensitive MEN were calbindin positive in our studies (Mazzuoli and Schemann, 2009; Kugler et al., 2015; Mazzuoli-Weber and Schemann, in review). These very likely were AH-IPANs because calbindin is a quite reliable marker for them, at least in the guinea pig ileum. In the study by Kunze and colleagues most of their impaled AH-IPANS were sensitive to compression (Kunze et al., 2000). However, this may not be a property of all calbindin or AH neurons.

## MEN responding to tensile forces

True tension-sensitive MEN were identified in the guinea pig distal colon (Spencer and Smith, 2004). In this study circumferential and longitudinal stretch activated so called S neurons (S because they receive fast synaptic input). These S neurons were distinct from AH-IPANs and were named mechanosensitive interneurons. Spiking in these tension-sensitive MEN did not cease when the muscle was paralyzed suggesting that they indeed respond to tensile forces. Based on the tracings illustrated in the publications tension-sensitive MEN belong to the population of USAMEN (Spencer and Smith, 2004). This group discovered that circumferential and longitudinal stretch activated different populations of MEN with similar firing behavior (Smith et al., 2007). Circumferential stretch activated ascending and descending cholinergic interneurons to activate excitatory and inhibitory muscle motor neurons and thereby mediating ascending contraction and descending inhibition of the muscle. In contrast, longitudinal stretch activated nitrergic interneurons to inhibit muscle activity. Tension-sensitive MEN with very similar characteristics to those in the colon also exist in the guinea pig stomach and ileum (Mazzuoli and Schemann, 2009; Mazzuoli-Weber and Schemann, in review). They were revealed by stretching individual ganglia by two hooks placed just next to the ganglion, thereby increasing the ganglionic area by only 3% (Mazzuoli-Weber and Schemann, in review). This was much less than the change in cross-sectional neuronal surface area observed in a maximally stretched or contracted tissue (Gabella and Trigg, 1984). About 27% of gastric myenteric neurons responded to stretch mostly with slowly adapting (44%) or ultra-slowly adapting (44%) spike discharge. Interestingly, most of them were nitric oxide synthase positive suggesting that they release nitric oxide upon activation.

Stretch-sensitive enteric neurons also exist in the esophagus. Cell swelling by applying hypotonic solution or stretching cultured neurons evoked long lasting Ca^2+^ increase together with nitric oxide release (Dong et al., 2015).

## MEN responding to compressive and tensile forces

There are only very few studies that directly address the question whether MEN may respond to several types of mechanical stress. From our own experiments we can conclude that about half of compression-sensitive MEN also respond to tensile forces (Mazzuoli-Weber and Schemann, in review). This conclusion is based on experiments where we recorded from MEN after distension of the ganglionic area and after intraganglionic volume injection. Measurements of the changes in ganglionic area during deformation indicated that the former stimulus would primarily mimic tensile forces while the latter one induces compressive forces. Fifty-three percent of MEN responded to both stimuli suggesting that the same neuron is able to sense different modes of mechanical stress. This finding was also supported by the fact that 46% of primary cultured MEN responded to tensile stress (pulling an elastic matrix) and compression (von Frey hair) (own unpublished results). Increase in tension caused slowly adapting firing while compression in the same neuron was associated with rapidly adapting firing. It remains to be studied whether MEN express structures specifically encoding tensile and compressive forces or whether the same mechanosensitive element is activated differently by compression and tension. It seems to be a general feature of MEN that responses to compressive stress adapt rapidly whereas, responses to tensile stress adapt slowly.

Intestinofugal neurons in the guinea pig colon responded to von Frey hair compression and circumferential tissue stretch (Hibberd et al., 2012a,b). Noteworthy, intestinofugal neurons in the mouse colon were activated by circumferential but not longitudinal stretch (Miller and Szurszewski, 2003). Similar to tension-sensitive MEN in the colon, their activation was independent from the degree of muscle tension (Hibberd et al., 2012a). This is puzzling because their sensitivity to von Frey hair stimulation would suggest that they also respond to tensile forces. Either the increase in muscle tension in the stretched preparation was too low or the firing adapted as a result of the stretch. Another example of visceral nerves responding to both types of mechanical stress are mechanosensitive extrinsic afferents which directly respond to distension of the gut wall as well as to compression by von Frey hairs (Zagorodnyuk and Brookes, 2000; Zagorodnyuk et al., 2001; Lynn et al., 2003, 2005; Grundy, 2004).

## Common features of MEN

Very early on it has been suggested that MEN provide simultaneous tonic input to both neurons and muscle fibers (Wood, 1970). In line with this, AH-IPANs as well as the uniaxonal mechanosensitive interneurons in the guinea pig myenteric plexus project to other neurons in the same plexus (Pan and Gershon, 2000; Spencer and Smith, 2004; Furness, 2006). Some compression-sensitive MEN were classified as circular muscle motor neurons because their long process can be traced to the circular muscle where it ramifies (Mazzuoli and Schemann, 2009). Most of the mechanosensitive esophageal neurons expressed nitric oxide synthase and the authors concluded that they also function as inhibitory motor neurons (Dong et al., 2015). It seems to be a general feature of MEN that they serve several functions; hence they have to be viewed as multifunctional neurons. A given mechanosensitive neuron has mechanosensitive processes and at the same time processes serving motor functions (Kugler et al., 2015).

All MEN receive numerous fast or slow synaptic inputs which tunes their excitability not only to other transmitters but also to mechanical stimulation (see Blackshaw et al., 2007). As a consequence mechanosensitivity in the ENS depends on low-precision sensors. This agrees with the finding that responses in RAMEN to repeated compressions evoked every time a rapidly adapting response despite the fact that the deformation from the previous compression persisted and gradually increased (Mazzuoli-Weber and Schemann, in review). This strongly suggests that compression-sensitive RAMEN do not encode the absolute degree of deformation but react every time the compressive forces change.

## Proportion of MEN

The exact proportion of mechanosensitive neurons in the ENS is not known but has certainly been underestimated so far. In our own studies, the proportion of compression-sensitive MEN varies between 14% in the mouse colon, 22% in the mouse ileum, 25% in the guinea pig ileum, and 27% in the guinea pig stomach. We assume these percentages include some of the tension-sensitive MEN (mechanosensitive interneurons) because their properties are similar (Spencer and Smith, 2004). Although, there is some minor overlap between MEN and AH-IPANs, they are basically different populations. In the guinea pig ileum AH-IPANs account for about 30% of all myenteric neurons. This means that up to 60% of all myenteric neurons respond to mechanical stimulation. It has to be noted that none of them function as sensory neuron in a classical sense. All MEN eventually turned out to be mechanosensitive inter or motor neurons. However, it is equally important to realize that not all interneurons or motor neurons are mechanosensitive.

## MEN as part of enteric reflexes controlling gut motility

**Figure 4:**
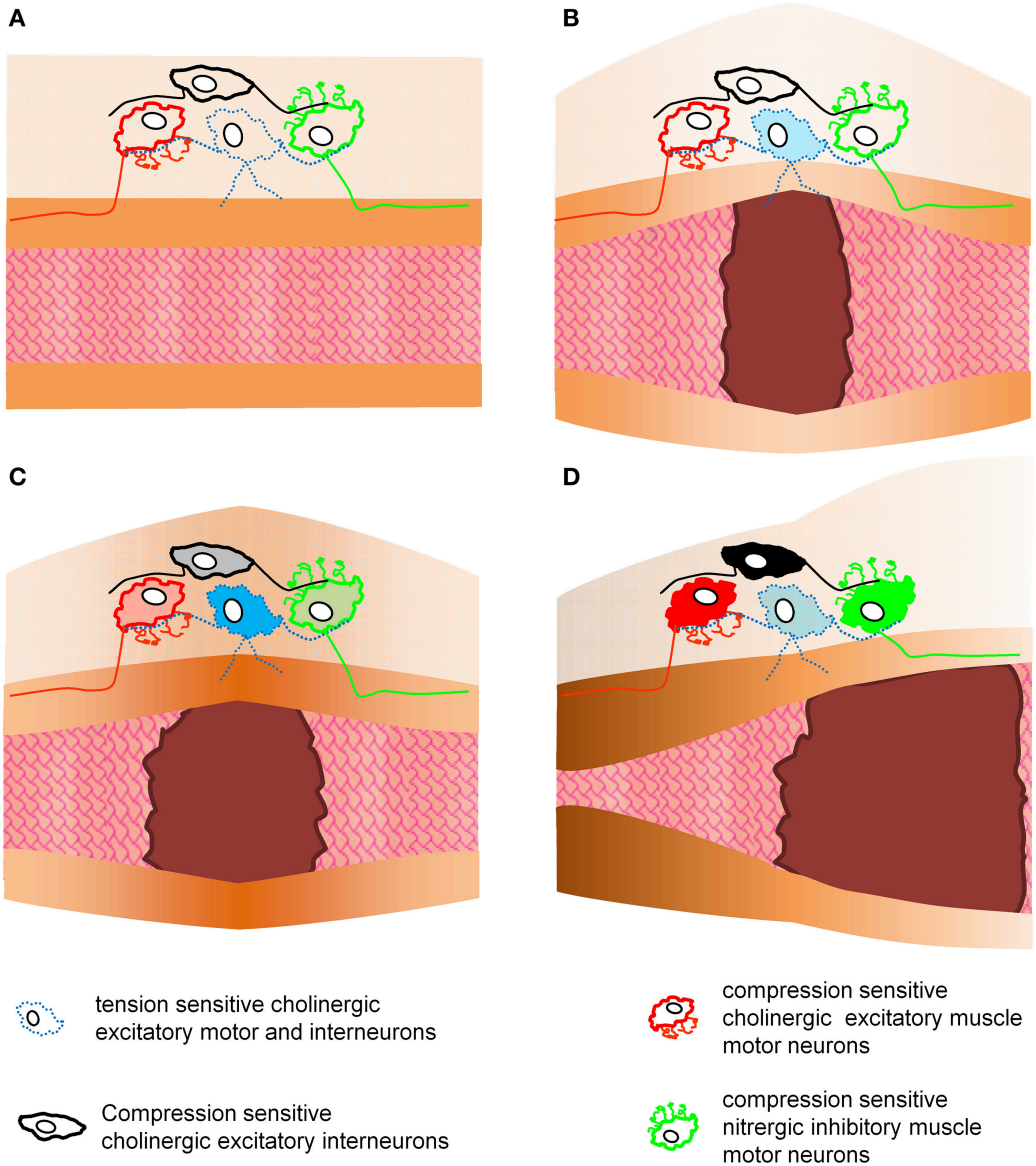
**This figure illustrates the putative roles of tension and compression sensitive motor and interneurons in enteric reflexes controlling muscle activity**. This is a simplified model as it only considers circumferential stretch and contractions. Panel **(A)** shows an empty gut region without mechanical stress acting on enteric neurons. In panel **(B)** a bolus distends the gut and causes activation of tension sensitive neurons. These neurons may act as interneurons and another population as motor neurons. Activation of motor neurons would cause an increase in muscle tone at the site of distension **(C)**. This again will trigger compressive sensitive interneurons or motor neurons to evoke proximal contraction and distal inhibition of the muscle **(D)**. It would be theoretically possible to evoke this enteric reflex without synaptic transmission because the motor neurons are tensile and compressive sensitive. The hexamethonium resistant reflex activity provides an indication that such a phenomenon may exist.

Figure 4 illustrates how compression and tension-sensitive MEN may be involved in enteric reflexes controlling muscle activity (Figure 4). Certainly compressive and tensile stress may trigger various motor patterns in the gastrointestinal tract. Phasic contractile activity prevails throughout the gut but longer lasting adaptations in muscle tone become important whenever the gut needs to accommodate different volumes. Basal muscle tone imposes tensile forces onto neurons and this resting tone provides ongoing input to compression-sensitive ultra-slowly adapting MEN. Direct evidence for this concept is the ongoing spike discharge in AH-IPANs (USAMEN) when muscle tone is maintained. Circumstantial evidence is the phenomenon that AH-IPANs are much more excitable in a stretched preparation with muscle tone compared to preparations at slack or treated with nifedipine (personal experience of M.S.). Phasic contractions throughout the gut last only for a few seconds. This may cause brief activation of compression-sensitive RAMEN. In particular the slowly and ultra-slowly adapting tension-sensitive MEN are candidates to control and respond to long lasting changes in muscle tone. For example in the stomach most of the ultra-slowly adapting tension-sensitive MEN have a nitrergic phenotype (Mazzuoli-Weber and Schemann, in review). Their firing behavior and their transmitter phenotype makes them likely candidates for initiating the decrease in muscle tone to facilitate adaptive volume accommodation. In other areas like the colon some ultra-slowly adapting tension-sensitive MEN may be responsible to generate tone in response to circumferential tissue stretch but to lower tone during longitudinal stretch (Smith et al., 2007).

Interestingly, AH-IPANs only fired when tissue stretch was associated with muscle tone. However, they were directly activated by compressive forces after von Frey hair probing (Kunze et al., 2000). This is not necessarily a contradiction as one can imagine that a process of an AH-IPAN is compressed if the distension is associated with an increase in muscle tone. The morphology of AH-IPANs is perfectly suited to react to increased tone of the circular muscle because most of their long processes run circumferential. However, it remains an important aspect that they do not respond to stretch alone. Responses in compression-sensitive MEN are independent of muscle tone yet muscle tone is probably responsible for the development of compressive forces under physiological conditions.

It is tempting to speculate that compression-sensitive MEN themselves regulate the forces they are exposed to: a servo-feedback loop. This is supported by their cholinergic or nitregric phenotype and their axonal projections to the circular muscle (Mazzuoli and Schemann, 2009; Mazzuoli-Weber and Schemann, in review) which upon activation would release acetylcholine or nitric oxide to contract or relax the muscle, respectively. Blocking the nicotinic receptors decreased or even abolished the distension evoked proximal contraction and distal inhibition of the muscle (Feldberg and Lin, 1949). This supports the important involvement of a nicotinic synapse in the enteric reflex pathway. In some studies the excitatory and inhibitory components recovered after a few minutes (Kosterlitz and Robinson, 1957), or were restored in naloxone (Barthó et al., 1989). This suggested that the compression-sensitive MEN which have projections to the circular muscle (mechanosensitive motor neurons; Mazzuoli and Schemann, 2009) were directly activated by the tension increase at the distended region. Since these neurons are cholinergic and nitrergic their ascending and descending projections regulate ascending contraction and descending inhibition of the muscle, respectively, without involvement of specialized mechanosensitive neurons or interneurons. As a prerequisite we suggest that the tension-sensitive cholinergic slowly and ultra-slowly adapting MEN directly increase muscle tone in the distended region. This would not require any synapse as tension and compression-sensitive motor neurons directly respond to the mechanical stress modalities. Alternatively, the tension-sensitive cholinergic slowly and ultra-slowly adapting MEN may drive locally projecting cholinergic motor neurons in order to increase muscle tone in the distended region. In this way peristalsis, i.e., spatiotemporally coordinated propagation of contractions, may be a result of a perpetual activation of tensile and compression-sensitive neurons without the need for synaptic transmission.

## Outlook

So far, neither the sensitivity and specificity of MEN to different forms of mechanical stress (compression or tension) nor their firing pattern (rapid, slow, or ultra-slow adaptation) has been considered in enteric reflex circuits. One of the biggest challenges is to incorporate compressive and tension-sensitive MEN into a neural pathway that controls muscle activity (Figure 4). MEN may interact to control motor patterns or various forms of a particular pattern. Alternatively, they may regulate independently from each other different motor patterns. As discussed above, there is evidence that different circuits are activated with distension of longitudinal or circular muscle (Smith et al., 2007). It is obviously important for colonic reflex activity whether the gut is elongated or circumferentially stretched. It seems plausible that this is also relevant in other gut regions but so far this has not been studied in the small intestine or stomach.

Another important aspect for future studies is the identification of the mechanosensitive channel(s) or receptor(s). The question to address then is whether compression and tension-sensitive MEN express different channels and how targeted pharmacological interventions affect motor patterns. There are too few studies to make firm conclusions but they produced some promising results. One possible mechanosensitive structure is the large–conductance (BK) potassium channels. Mechanical deformation by increasing intraelectrode pressure increased open probability of BK channels (Kunze et al., 2000). This, however, would only explain the inhibition of neuronal activity after soma distortion; a phenomenon so far only reported once in AH-IPANs. Nevertheless, BK channels remain interesting candidates as they may exist in two variants. Studies on stretch induced extra systoles in chick heart suggested that myocytes expressed besides the non-stretch-sensitive BK channels also stretch-activated BK channels (Iribe et al., 2011). Other commonly used blockers of stretch-activated channels, such as 2-aminoethoxydiphenyl borate or *Grammostola* mechanotoxin 4, efficiently attenuated the swelling induced Ca^2+^ increase in cultured enteric neurons from rat esophagus (Dong et al., 2015). The pharmacological approach is problematic as many blockers used are not specific for mechanosensitive channels. Moreover, there are no blockers available for some mechanosensitive channels, in particular for those that are directly gated by changes in lipid bilayer or cytoskeletal architecture. An alternative approach is to knock out the candidate channel bearing the problem that negative results may simply suggest compensation by other channels or receptors.

Finding answers to these open questions will not only advance basic knowledge on mechanosensitivity in the gut but will greatly advance the options for more specific treatments of the many gastrointestinal disorders caused by or associated with sensory dysfunctions at the level of the ENS.

## Funding

This work is supported by the German Research Foundation DFG (MA-5202/1-1 and 1-2) and the Technische Universität München within the funding program Open Access Publishing.

### Conflict of interest statement

The authors declare that the research was conducted in the absence of any commercial or financial relationships that could be construed as a potential conflict of interest.

## Supplementary material

Movie 1 **This slow motion movie (original length is 2.0 s) shows a primary cultured mechanosensitive enteric neuron (MEN) firing action potentials after neurite stimulation with a von Frey hair**. The white (originally red) outline of the neuron can be seen because Di-8-ANEPPS incorporates into the membrane. The stimulation site is marked with the yellow arrowhead. The trace at the bottom shows the spike discharge in this particular MEN. Once the movie starts there is a running bar indicating the elapsed time. The bar is running from 0 to 2.0 s. The overlay illustrates the color coded signals from the MEN; red color indicates discharge of action potentials. Shortly after the stimulus the neuron fired action potentials (red color). The firing adaptation defines the neuron as a slowly adapting MEN (SAMEN).
